# Evaluation of EBV- and HCMV-Specific T Cell Responses in Systemic Lupus Erythematosus (SLE) Patients Using a Normalized Enzyme-Linked Immunospot (ELISPOT) Assay

**DOI:** 10.1155/2019/4236503

**Published:** 2019-02-17

**Authors:** Irene Cassaniti, Lorenzo Cavagna, Sandra A. Calarota, Kodjo Messan Guy Adzasehoun, Giuditta Comolli, Carlomaurizio Montecucco, Fausto Baldanti

**Affiliations:** ^1^Molecular Virology Unit, Microbiology and Virology Department, Fondazione IRCCS Policlinico San Matteo, Pavia, Italy; ^2^Rheumatology Department, Fondazione IRCCS Policlinico San Matteo, Pavia, Italy; ^3^Experimental Research Laboratories, Biotechnology Area, Fondazione IRCCS Policlinico San Matteo, Pavia, Italy; ^4^Department of Clinical, Surgical, Diagnostic and Pediatric Sciences, University of Pavia, Pavia, Italy

## Abstract

Systemic lupus erythematosus (SLE) is an autoimmune disease with a complex etiology. Opportunistic viral pathogens, such as human cytomegalovirus (HCMV) and Epstein-Barr virus (EBV), are particularly relevant. The role of the T cell response in SLE has not been deeply studied; we investigated the role of HCMV- and EBV-specific T cell responses in SLE patients also in relation to their pharmacological immunosuppressive status. PBMCs from 70 SLE patients and 50 healthy controls were stimulated with EBV- and HCMV-specific antigens, and IFN-*γ*-secreting T cells were quantified. We observed that both EBV- and HCMV-specific T cell responses were significantly lower in SLE patients compared with healthy subjects. We reported decreased EBV- and HCMV-specific T cell responses among medium-high immunosuppressed patients compared to low immunosuppressed patients. Immunosuppressive level could exert a role in the control of herpesviruses reactivation, even if the immunosuppressive condition of SLE remains the driving cause of skewed virus-specific T cell response.

## 1. Introduction

Systemic lupus erythematosus (SLE) is a complex pathological condition [1] which may be considered the prototypical example of systemic autoimmune disease [2]. SLE is characterized by a wide range of clinical manifestations, with variable degrees of severity and different profiles of autoantibody expression [2–6]. In more detail, persistent inflammatory state, detrimental for multiple organs, is typical of SLE. The clinical manifestation is influenced by ethnicity, gender, age, and socioeconomic factors [6]. However, even though clinical and laboratory heterogeneity is typical of SLE, immunosuppression, which is linked to both disease activity and treatment approaches, is the hallmark of the disease [7, 8]. In fact, due to this immunosuppression, SLE patients are exposed to increased risk of infections, deeply influencing their prognosis [9]. Among different pathogens, opportunistic viral infections such as human cytomegalovirus (HCMV) and Epstein-Barr virus (EBV) are particularly relevant [10]. These viruses belong to the *Herpesviridae* family, and they may complicate the disease course [11–15] or mimic several features of SLE [9]. Furthermore, both HCMV and EBV may induce disease flares and have been indicated in SLE pathogenic processes [15–21]. It has been proposed that EBV may induce SLE through molecular mimicry with the cross reaction of Epstein-Barr nuclear antigen 1 (EBNA-1) with self-antigens [22, 23], while HCMV is thought to cause autoimmunity through molecular mimicry, epitope spreading, and an induced immune response to cryptic antigens [24]. In this setting, the role of the immune system against opportunistic infections is crucial. Cell-mediated immunity is fundamental in the control of herpesviruses infections; interferon-gamma (IFN-*γ*) is also suggested to play a crucial role in this context [25].

The aim of this study was to evaluate and characterize T cell responses to HCMV and EBV in SLE patients using IFN-*γ* ELISPOT assay. By using a novel approach we provided a good estimation of both CD4^+^ and CD8^+^ antigen-specific T cell responses, avoiding predepletion assay [26]. In this way, it is possible to estimate the role of CD4^+^ and CD8^+^ antigen-specific T cell response, avoiding the intracellular cytokine staining approach that is labor intensive and requires a larger number of cells. However, this approach cannot be considered as precise as flow cytometry strategy, but could represent an easier way for the estimation of antigen-specific T cell response. For comparison, T cell response to the nonspecific mitogen (PHA) was also investigated.

## 2. Materials and Methods

### 2.1. SLE Patients and Healthy Controls

Seventy patients (64 females and 6 males, median age 46.5 years, interquartile range (IQR) 38.0-57.8) fulfilling the 1997 ACR classification criteria for SLE [27] and referred to the Rheumatology Division of the Fondazione IRCCS Policlinico San Matteo, University of Pavia, Italy, were included in this study. The study was approved by the Institutional Review Board (IRB) and all subjects, as well as the fifty healthy subjects (35 females and 15 males, median age 44 years, IQR 34.8-50.0) who were evaluated as controls, gave their written informed consent.

SLE patients had a median age at disease onset of 30 (IQR 23-46) years and a median disease duration of 121.5 (IQR 42.3-228.5) months. In all cases, disease activity was evaluated according to SLEDAI 2k score [28]. All patients had received stable treatment in the previous six months, and treatment regimens were registered in all cases.

For practical purposes, we divided the patients into two groups, according to the degree of pharmacological immunosuppression: patient treatment with hydroxychloroquine and/or with prednisone ≤ 5 mg/day was considered low pharmacological immunosuppression (lp-IS, no. of patients: 25). Patient treatment with mycophenolate mofetil, methotrexate, cyclosporin A, rituximab, belimumab, and/or prednisone > 5 mg/day was considered medium-high pharmacological immunosuppression (mhp-IS, no. of patients: 45).

### 2.2. Peripheral Blood Samples

Peripheral blood was collected into vacutainer tubes (BD) containing heparin. Whole blood was used for viral genome quantification and determination of T cell subsets; plasma was separated for serological analyses. Peripheral blood mononuclear cells (PBMCs) were isolated by density gradient centrifugation (Lymphoprep, Axis-Shield, Oslo, Norway), cryopreserved in freezing medium (65% RPMI 1640 supplemented with 2 mM L-glutamine, 100 U/ml penicillin and 100 *μ*g/ml streptomycin, 25% human albumin (Grifols Biologicals, Los Angeles, CA, USA), and 10% DMSO (Sigma-Aldrich, St. Louis, MO, USA)), and stored in liquid nitrogen (10 × 10^6^ cells/ml) until analysis. After thawing, about 50-60% of cells were still viable and could be used in the ELISPOT assay.

### 2.3. EBV-Specific Antibodies

IgM antiviral capsid antigen (anti-VCA), IgG anti-VCA, and anti-EBNA were quantified using an ELISA kit (DiaSorin, Vercelli, Italy), according to the manufacturer's instructions. Healthy subjects and SLE patients were considered EBV-seropositive when IgG anti-VCA and anti-EBNA were positive.

### 2.4. HCMV-Specific Antibodies

IgM anti-HCMV and IgG anti-HCMV were quantified using an ELISA kit (DiaSorin, Vercelli, Italy), according to the manufacturer's instructions. Healthy subjects and SLE patients were considered HCMV-seropositive when IgG anti-HCMV was positive.

### 2.5. Viral DNA Quantification

DNA was purified using NucliSENS®easyMAG® (bioMérieux, Lyon, France). EBV DNA and HCMV DNA were quantified using real-time PCR (lower limit detection HCMV and EBV DNA 90 copies/ml), as previously described (Baldanti et al. [29]; Furione et al. [30]).

### 2.6. T Cell Subsets

Fresh whole blood was stained with anti-CD3-PC5, anti-CD45-FITC, anti-CD4-RD1, and anti-CD8-ECD monoclonal antibodies (CYTO-STAT tetraCHROME; Beckman Coulter, Milan, Italy). After lysis of red blood cells, absolute CD3^+^, CD3^+^CD4^+^, and CD3^+^CD8^+^ T cell counts (cells/*μ*l) were determined by flow cytometry (Navios, Beckman Coulter), using Flow-Count Fluorospheres. Gating strategy was set up on CD45^+^ and side scatter (SSC).

### 2.7. Synthetic Peptides

Lyophilized peptide pools, 15 amino acids in length with an 11 amino acid overlap, were resuspended in RPMI 1640 supplemented with 2 mM L-glutamine, 100 U/ml penicillin, and 100 *μ*g/ml streptomycin with 8% of DMSO. Resuspended peptide pools were used after dilution (1 : 100) in RPMI 1640 supplemented with 2 mM L-glutamine, 100 U/ml penicillin and 100 *μ*g/ml streptomycin, and 10% FBS and then used as antigens. A lytic pool, containing peptides spanning the full length of BZLF-1 (59 peptides) and BMRF-1 (99 peptides) EBV proteins; an Epstein-Barr nuclear antigen (EBNA) pool, containing peptides spanning the full length of EBNA 1 (158 peptides), EBNA3a (234 peptides), EBNA 3b (279 peptides), and EBNA 3c (265 peptides) EBV proteins; and a latent membrane protein (LMP) pool, containing peptides spanning the full length of LMP1 (94 peptides) and LMP2 (122 peptides) EBV proteins, were used as EBV-specific antigens (JPT Peptide Technologies, Berlin, Germany) at a final concentration of 0.25 *μ*g/ml for each individual peptide in the corresponding pool. Peptide pools representative of whole HCMV proteins IE-1 (120 peptides), IE-2 (143 peptides), and pp65 (138 peptides) (JPT Peptide Technologies) were used at a final concentration of 0.25 *μ*g/ml for each individual peptide in the corresponding pool.

### 2.8. ELISPOT Assay

Human IFN-*γ* ELISPOT kits (Diaclone, Besancon, France) and Multiscreen-IP membrane-bottomed 96-well plates (Merck Millipore, Darmstadt, Germany) were used as described [26–31]. Briefly, plates were coated overnight with monoclonal capture antibody against IFN-*γ* and stored at 4°C. After washing with PBS, plates were blocked with culture medium (RPMI 1640 supplemented with 2 mM L-glutamine, 100 U/ml penicillin and 100 *μ*g/ml streptomycin, and 10% heat inactivated fetal bovine serum (FBS) (Euroclone)) for 2 hours at room temperature. Cells were plated in duplicate (1 × 10^5^/100 *μ*l per well) and stimulated with the corresponding antigens or with phytohemagglutinin (PHA, 5 *μ*g/ml, Sigma-Aldrich) or with medium alone (negative control) and incubated at 37°C in a 5% CO_2_ humidified atmosphere for 24 hours. After washing, plates were incubated overnight at 4°C with biotinylated IFN-*γ* detection antibody. Plates were washed, streptavidin-alkaline phosphatase conjugate was added, and plates were incubated at 37°C in a 5% CO_2_ atmosphere for 1 hour. Plates were then washed, and 5-bromo-4-chloro-3-indolyl phosphate/nitro blue tetrazolium (BCIP/NBT) was added for 20 minutes at room temperature. Wells were then washed several times under running water and air-dried overnight. Spots were counted by using an automated AID ELISPOT reader system (Autoimmun Diagnostika GmbH, Strasburg, Germany). The mean number of spots from duplicate wells was adjusted to 1 × 10^6^ PBMCs. The net spots per million PBMCs was calculated by subtracting the number of spots responding to the negative control from the number of spots responding to the corresponding antigenand results were given as net spots/million PBMCs. Furthermore, results were normalized to absolute CD4^+^ and CD8^+^ T cell counts, as previously described [31].

### 2.9. Statistics

Descriptive data were reported or considered as absolute and relative frequencies, mean and standard deviation, median, and interquartile range (IQR) based on the type of the variable distribution. For qualitative variables, Fisher's test was used, while *t*-test or Mann-Whitney test was used for quantitative variables in order to perform comparison between groups. Spearman's test was used for the correlation analysis. All tests were two-tailed. A *P* value < 0.05 was considered statistically significant. Analyses were performed using the GraphPad Prism 5 (GraphPad Software, CA, USA).

## 3. Results

### 3.1. T Cell Subsets, Serology, and Viral Load

T cell subsets were analyzed in 70 SLE patients and 50 healthy subjects with homogeneous characteristics. The two groups were not substantially different in terms of age and sex distribution. Both CD4^+^ and CD8^+^ T cells were significantly lower in SLE patients than in healthy controls (*P* < 0.0001). In SLE patients, the median CD4^+^ and CD8^+^ T cell counts were 434.5 (IQR 270.3-620.3) cells/*μ*l and 287.0 (IQR 196.0-397.3) cells/*μ*l, respectively, while in healthy subjects, the median CD4^+^ and CD8^+^ T cell counts were 1054.0 (IQR 760.5-1361.0) cells/*μ*l and 532.0 (IQR 376.8-748.8) cells/*μ*l, respectively. EBV- and HCMV-specific antibodies were analyzed in all enrolled subjects, as well as HCMV and EBV DNAemia. Demographic data are summarized in Table 1.

### 3.2. Measurement of EBV-Specific T Cell Responses

EBV-specific CD4^+^ and CD8^+^ T cell responses to all EBNA, LMP, and lytic overlapping 15-mer peptide pools were evaluated in 68 EBV-seropositive SLE patients, 25 EBV-seropositive healthy subjects, and four EBV-seronegative individuals (2 SLE patients and 2 healthy subjects).

**Figure 1:**
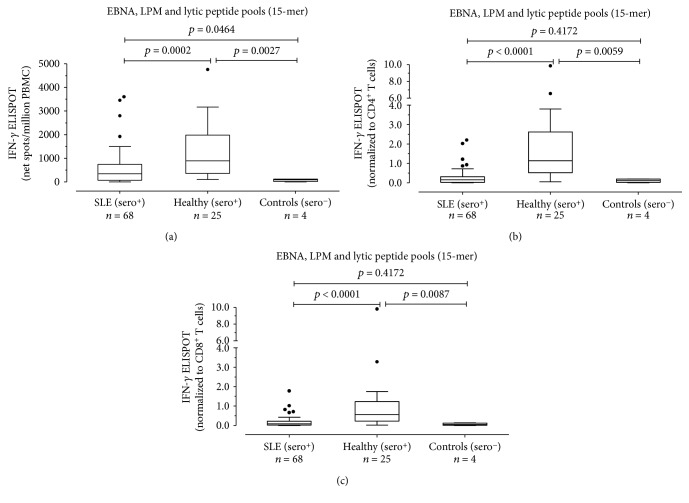
PBMCs from 68 EBV-seropositive SLE patients (SLE (sero+)), 25 EBV-seropositive healthy subjects (healthy (sero+)), and 4 EBV-seronegative controls (controls (sero-)) were evaluated in response to EBNA, LMP, and lytic peptide pools (15 amino acids in length with an 11 amino acid overlap), and results were given as net spots/million PBMCs (a) and normalized to CD4^+^ and CD8^+^ T cell counts. Results are also presented as EBV-specific IFN-*γ*^+^ CD4^+^ (b) or CD8^+^ (c) T cells/*μ*l. *P* values were calculated with the Mann-Whitney test.

Median EBV-specific T cell response was significantly lower in EBV-seropositive SLE patients than in EBV-seropositive healthy subjects (340.5 (IQR 75-738.8) vs. 890 (IQR 360-1983) net spots/million PBMCs; *P* = 0.0002) (Figure 1(a)). Normalizing results to CD4 and CD8 T cell counts, we observed that EBV-specific CD4^+^ T cell response was lower in EBV-seropositive SLE patients than in EBV-seropositive healthy subjects (0.1530 (IQR 0.02325-0.3145) vs 1.140 (IQR 0.5235-2.6180) EBV-specific CD4^+^ T cells/*μ*l EBV-specific CD4^+^ T cells/*μ*l; *P* < 0.0001) (Figure 1(b)). Similarly, EBV-specific CD8^+^ T cell response was significantly different in the two groups of subjects (0.0905 (IQR 0.0215-0.2148) and 0.5620 (IQR 0.2335-1.231) EBV-specific CD8^+^ T cells/*μ*l, respectively, *P* < 0.0001) (Figure 1(c)). There was no correlation between EBV-specific CD4^+^ and CD8^+^ T cell responses with the SLEDAI 2k score in SLE patients (data not shown).

**Figure 2:**
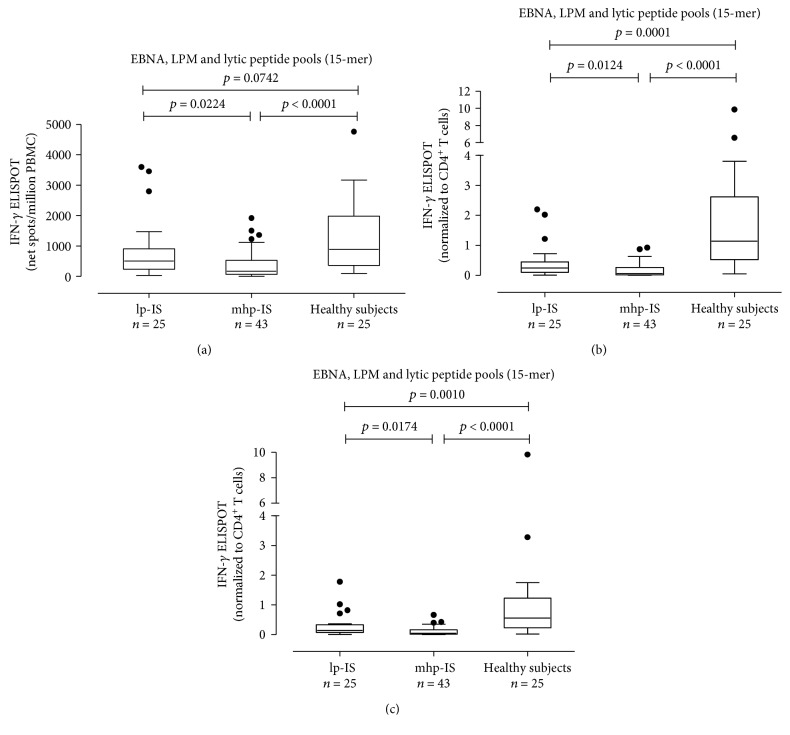
EBV-specific T cell response as net spots/million PBMCs (a) and EBV-specific CD4^+^ (b) and CD8^+^ (c) T cell responses in SLE patients treated with low immunosuppression (lp-IS, *n* = 25) and SLE patients with medium-high immunosuppression (mhp-IS, *n* = 43) are presented. *P* values were calculated with the Mann-Whitney test.

EBV-specific T cell responses were analyzed in patients receiving lp-IS (*n* = 25, 36.8%) or mhp-IS (*n* = 43, 63.2%). The latter had significantly lower EBV-specific T cell response than the other group (170 (IQR 70-530) and 500 (IQR 237.5-905.0) net spots/million PBMCs, respectively; *P* = 0.0224) (Figure 2(a)). Results were normalized to CD4^+^ and CD8^+^ T cells. The EBV-specific CD4^+^ T cell response was lower in mhp-IS than in lp-IS (0.0620 (IQR 0.0170-0.2680) and 0.2420 (IQR 0.1065-0.4515) EBV-specific CD4^+^ T cells/*μ*l; *P* = 0.0124) (Figure 2(b)). Similarly, the CD8^+^ T cell response in SLE patients receiving mhp-IS was lower than that observed in patients receiving lp-IS (0.1430 (IQR 0.0800-0.3335) and 0.0450 (IQR 0.0180-0.1640) EBV-specific CD8^+^ T cells/*μ*l; *P* = 0.0174) (Figure 2(c)).

**Figure 3:**
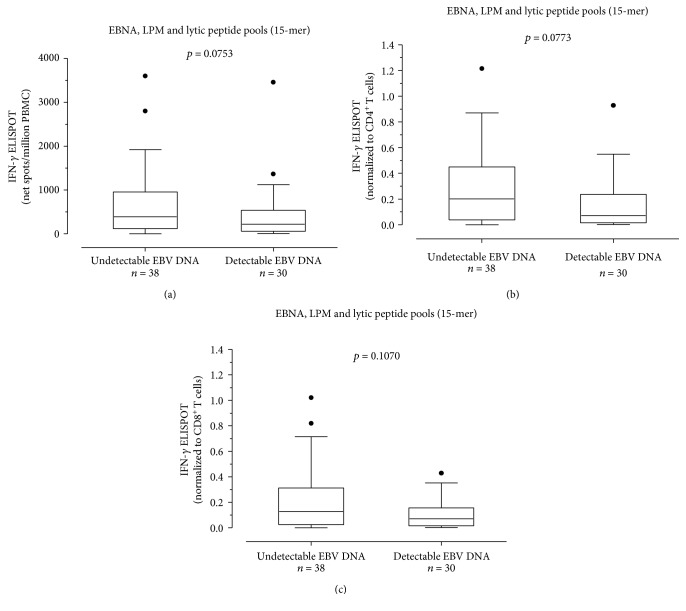
EBV-specific T cell response as net spots/million PBMCs (a) was evaluated in patients with undetectable EBV DNA (*n* = 38) and detectable EBV DNA (*n* = 30). Results were also normalized to CD4^+^ T cell (b) and CD8^+^ T cell (c) and presented as EBV-specific CD4^+^ or CD8^+^ IFN-*γ*^+^ T cells/*μ*l. *P* values were calculated with the Mann-Whitney test.

Additionally, we evaluated the CD4^+^ and CD8^+^ T cell EBV-specific ELISPOT response in SLE patients with undetectable EBV DNA (*n* = 38; 55.9%) and detectable EBV DNA (*n* = 30; 44.1%). A trend toward statistical significance was observed when we compared the EBV-specific T cell response measured as net spots/million PBMCs in SLE patients with undetectable (median 387.5 (IQR 119.8-953.8)) and detectable EBV DNA (220 (IQR 56.3-538.8); *P* = 0.0753) (Figure 3(a)). Similarly, a trend toward statistical significance was observed comparing median EBV-specific CD4^+^ T cells/*μ*l between the two groups (0.202 IQR (0.038-0.450) and 0.072 IQR (0.016-0.236) EBV-specific CD4^+^ T cells/*μ*l; *P* = 0.0773) (Figure 3(b)), although no difference was observed in terms of EBV-specific CD8^+^ T cell response (respectively, median 0.128 IQR (0.0245-0.3115) EBV-specific CD8^+^ T cells/*μ*l and 0.0710 IQR (0.0157-0.1565) EBV-specific CD8^+^ T cells/*μ*l; *P* = 0.1070) (Figure 3(c)).

**Figure 4:**
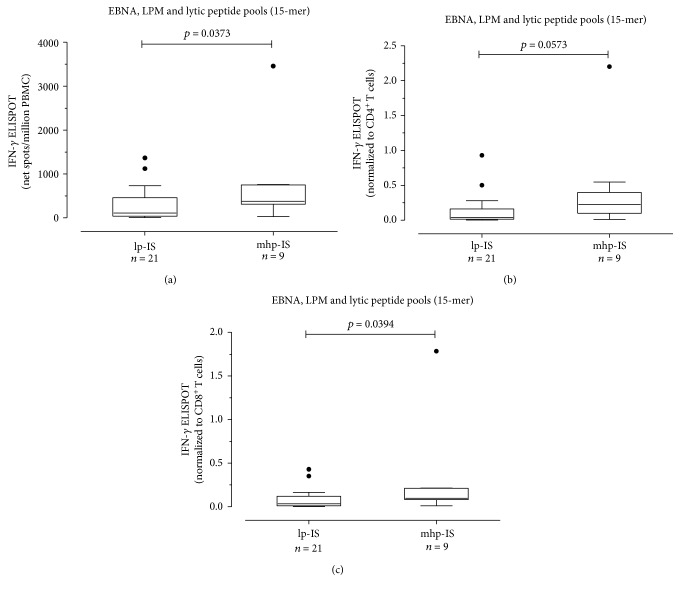
EBV-specific T cell response in SLE patients with detectable EBV DNA was measured. Patients were classified in medium-high immunosuppressed (mhp-IS; *n* = 21) and low immunosuppressed (lp-IS *n* = 7) patients. Results were given as net spots/million PBMCs (a) and then normalized on CD4^+^ (b) and CD8^+^ (c) T cell count. *P* values were calculated with the Mann-Whitney test.

Among the 30 SLE patients with detectable EBV DNA, 21 (70%) were receiving mhp-IS and nine (30%) lp-IS. In a comparison of the two groups, EBV-specific T cell response measured as net spots/million PBMCs was higher in lp-IS group (median 110 IQR (37.5-460)) than in mhp-IS (median 380 IQR (313-750); *P* = 0.0373) (Figure 4(a)). A trend toward statistical significance was observed when the EBV-specific CD4^+^ T cell response in mhp-IS patients (median 0.040 IQR (0.013-0.160) EBV-specific CD4^+^ T cells/*μ*l) was compared with lp-IS patients (median 0.223 IQR (0.097-0.398) EBV-specific CD4^+^ T cells/*μ*l; *P* = 0.0573) (Figure 4(b)). A significant difference was found between the CD8^+^ T cell response in mhp-IS (0.034 IQR (0.011-0.120) EBV-specific CD8^+^T cells/*μ*l and lp-IS patients (0.097 IQR (0.080-0.210) EBV-specific CD8^+^ T cells/*μ*l; *P* = 0.0394) (Figure 4(c)). No difference was found in terms of EBV-specific T cell response in SLE patients with undetectable EBV DNA classified according to immunosuppression level.

### 3.3. Measurement of HCMV-Specific CD4^+^ and CD8^+^ T Cell Responses

**Figure 5:**
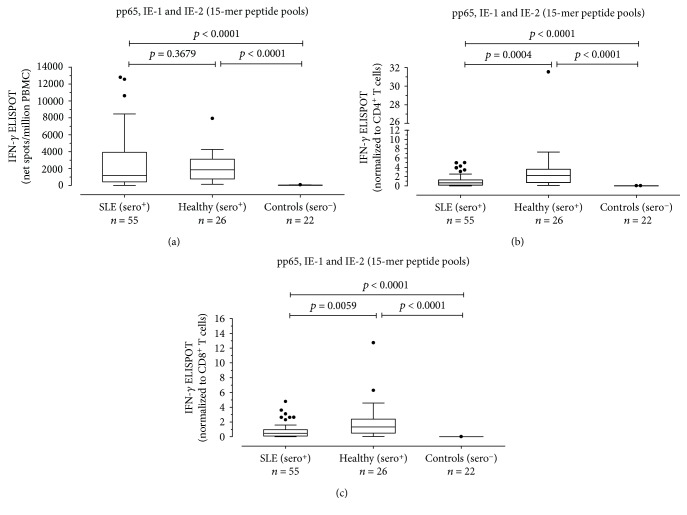
PBMCs from 55 HCMV-seropositive SLE patients (SLE (sero+)), 26 HCMV-seropositive healthy controls (healthy (sero+)), and 22 HMCV-seronegative controls (controls (sero-)) were evaluated in response to pp-65, IE-1, and IE-2 peptide pools (15 amino acids in length with an 11 amino acid overlap). Results were given in terms of net spots/million PBMCs (a) and then normalized to CD4^+^ (b) and CD8^+^ (c) T cell counts. *P* values were calculated with the Mann-Whitney test.

**Figure 6:**
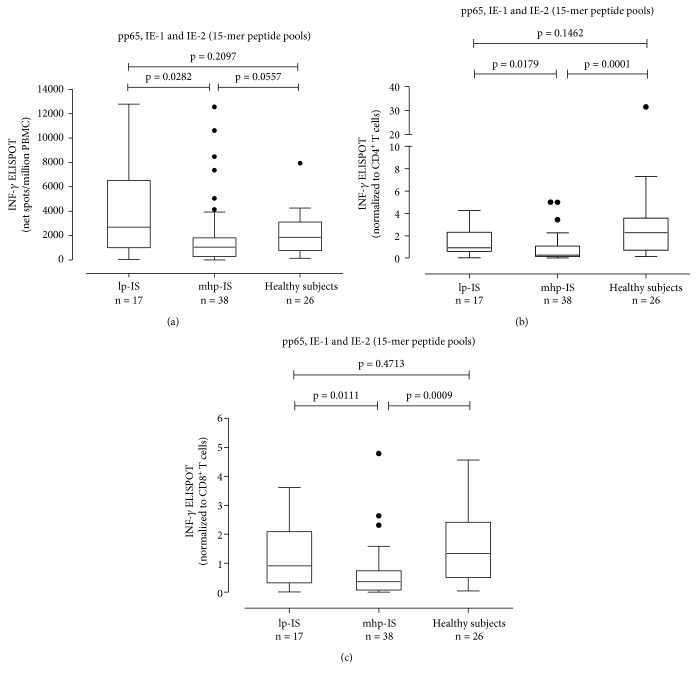
HCMV-specific T cell response was measured in SLE patients receiving low immunosuppression (lp-IS, *n* = 17) and medium-high immunosuppression (mhp-IS, *n* = 38). Results were presented as net spots/million PBMCs (a) and normalized to CD4^+^ (b) and CD8^+^ (c) T cell responses. *P* values were calculated with the Mann-Whitney test.

HCMV-specific T cell response to pp65, IE-1, and IE-2 overlapping 15-mer peptide pools was evaluated in 55 HCMV-seropositive SLE patients, 26 HCMV-seropositive healthy subjects, and 22 HCMV-seronegative controls (14 SLE patients and 8 healthy subjects). HCMV-specific T cell response was lower in HCMV-seropositive SLE patients than in HCMV-seropositive healthy subjects (median 1155 (IQR 415-3925) and 1850 (770-3103) net spots/million PBMCs, respectively), but the difference was not statistically significant (*P* = 0.3679) (Figure 5(a)). Results were normalized to CD4^+^ and CD8^+^ T cell count. The HCMV-specific CD4^+^ T cell response was significantly lower in HCMV-seropositive SLE patients compared to HCMV-seropositive healthy subjects (0.2700 (IQR 0.0630-0.9200) vs 0.6065 (IQR 0.1368-1.250) HCMV-specific CD4^+^ T cells/*μ*l; *P* = 0.0004) (Figure 5(b)). Similarly, the HCMV-specific CD8^+^ T cell response was significantly different in the two groups of subjects (0.6490 (IQR 0.1480-1.280) vs 2.279 (IQR 0.7108-3.570) HCMV-specific CD8^+^T cells/*μ*l; *P* = 0.0059). Both groups of HCMV-seropositive subjects (SLE patients and healthy subjects) had HCMV-specific responses higher than HCMV-seronegative controls (*P* < 0.0001) (Figure 5(c)). There was a negative correlation between the HCMV-specific CD4^+^ T cell response and SLEDAI 2k score, but no correlation was found between the HCMV-specific CD8^+^ T cell response and SLEDAI 2k score (data not shown). Seventeen out of 55 (30.9%) SLE patients were receiving lp-IS, while the remaining 38 (69.1%) were receiving mhp-IS. The HCMV-specific ELISPOT response measured as net spots/million PBMCs was significantly higher in lp-IS patients (median 2695 (IQR 1003-6525)) than in mhp-IS patients (median 1055 (IQR 271-1810)) (*P* = 0.0282) (Figure 6(a)). Similarly, the HCMV-specific CD4^+^ T cell response was significantly reduced in this mhp-IS patients (0.271 IQR (0.125-1.083) vs 0.927 IQR (0.611-2.315) HCMV-specific CD4^+^ T cells/*μ*l; *P* = 0.0179) (Figure 6(b)). Finally, there was a significant difference in terms of HCMV-specific CD8^+^ T cell response between mhp- and lp-IS (0.359 IQR (0.071-0.738) vs 0.910 IQR (0.383-2.090) HCMV-specific CD8^+^ T cells/*μ*l; *P* = 0.0111) (Figure 6(c)).

We observed that ELISPOT T cell response in the 52 SLE patients with undetectable HCMV DNA was higher than the response observed in the three patients with detectable HCMV DNAemia (data not shown). However, due to the low number of patients in the latter group, the difference was not considered statistically valid.

All patients with detectable HCMV DNA were medium-high immunosuppressed. Two of them were also positive for EBV DNA.

### 3.4. Measurement of T Cell Response to PHA

**Figure 7:**
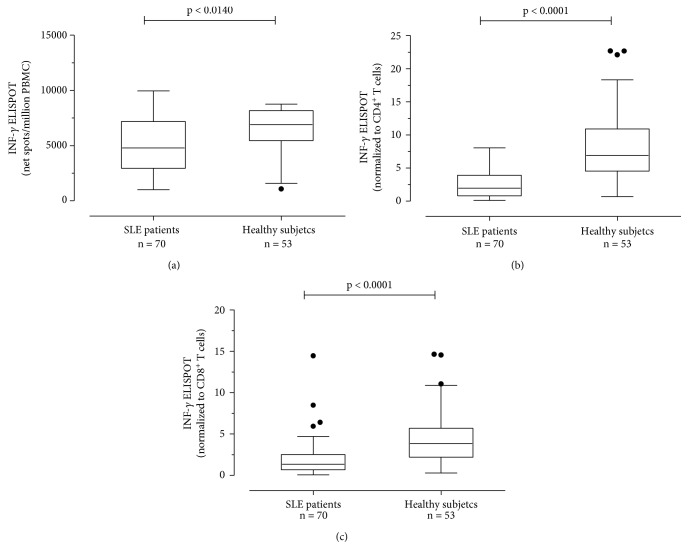
PBMCs from 70 SLE patients and 53 healthy subjects were evaluated in response to PHA. Results were given as net spots/million PBMCs (a) and then normalized to CD4^+^ (b) and CD8^+^ (c) T cell counts. *P* value was calculated with the Mann-Whitney test.

CD4^+^ and CD8^+^ T cells producing IFN-*γ* in response to PHA, a nonspecific antigen, were significantly lower in 70 SLE patients than in 41 healthy controls. PHA-specific T cell responses measured as net spots/million PBMCs were 4795 (IQR 2956-7193) and 6915 (5449-8165) (*P* = 0.0140), respectively, (Figure 7(a)). Responses were normalized to CD4^+^ and CD8^+^ T cell count. Normalized PHA-specific CD4^+^ T cell responses were 1.978 (IQR 0.8085-3.927) and 6.890 (IQR 4.542-10.89) PHA-specific CD4^+^ T cells/*μ*l (*P* < 0.0001), respectively, (Figure 7(b)). Normalized PHA-specific CD8^+^ T cell responses were, respectively, 1.347 (IQR 0.6865-2.516) and 3.859 (IQR 2.199-5.700) PHA-specific CD8^+^T cells/*μ*l (*P* < 0.0001) (Figure 7(c)). There was no correlation between PHA response and SLEDAI score. Similarly, there was no difference in terms of PHA-specific T cell response according to immunosuppressive regimen.

## 4. Discussion

The aim of this study was to evaluate EBV- and HCMV-specific T cell responses by ELISPOT assay. In this setting, we used the normalization on CD4 and CD8 T cells in order to provide an estimation of CD4^+^ and CD8^+^ T cells producing IFN-*γ*, as previously described [26]. As our knowledge, the IFN-*γ* ELISPOT is one of the most widespread assays used to evaluate antigen-specific T cell response. It allows the evaluation of total CD4^+^ and CD8^+^ antigen-specific T cell response by detection of IFN-*γ*-producing T cells. As known, IFN-*γ* is a cytokine mainly produced by activated T helper 1 (Th1) and T cytotoxic cells in response to specific antigens, having a crucial role inactivating lymphocytes to enhance antimicrobial and antitumor effects. The traditional ELISPOT assay does not easily distinguish between CD4^+^ and CD8^+^ T cell responses unless separate antigens to detect CD4^+^ and CD8^+^ T cell responses or lymphocyte subset depletion are used [32]. EBV- and HCMV-specific peptide pools of 15 amino acids in length with an 11 amino acid overlap were used as stimuli. Such peptide pool represents a good compromise for stimulating both CD4^+^ and CD8^+^ T cells [33, 34]. Results were normalized to CD4^+^ and CD8^+^ T cell counts, in order to estimate both CD4^+^ and CD8^+^ T cells producing IFN-*γ*, in response to different viral antigens. Even if it does not allow a precise quantification of CD4^+^ and CD8^+^ antigen-specific T cell response, our previous results evidenced a good correlation between intracellular cytokine staining and normalized ELISPOT approach [26].

Among viral pathogens, EBV infection is the most common in patients with SLE, and more importantly, it has been hypothesized that EBV may play a role in SLE disease induction [34]. In a study by James et al., the incidence of EBV DNAemia was reported as similar in SLE patients and healthy controls (95% vs 99.5%) [35], while in two other studies, they reported that the prevalence of EBV infection was significantly higher in young SLE patients than in controls (99.6% vs 70%) [36, 37]. We observed an increased percentage of detectable EBV DNA among EBV-seropositive SLE patients compared with controls. Nevertheless, there was no difference in terms of median viral load between the two groups. Interestingly, 70% of SLE patients with detectable EBV DNA were treated with mhp-IS. This result could be suggestive of the involvement of immunosuppressive therapy in the control of reactivation. However, the difference between low immunosuppressed and medium-high immunosuppressed patients in terms of EBV-specific T cell response is low. Even if immunosuppressive treatments seem to exert a role in reduced EBV-specific T cell response, in this setting, there is an innate feature of SLE patients to have reduced immunity to EBV.

Both CD4^+^ and CD8^+^ T cell responses to EBV-specific peptide pools were significantly lower in SLE patients compared with controls. Berner et al. demonstrated that the frequency of EBV-specific CD8^+^ T cells was similar between SLE and healthy subjects when analyzed by using MHC I tetramers with a lytic cycle EBV antigen peptide. However, CD8^+^ T cell EBV-specific IFN-*γ* production was significantly lower in SLE patients than in healthy controls, when assayed by ELISPOT [38]. Nevertheless, no information regarding the CD4^+^ T cell EBV-specific response was provided. In contrast with our results, Kang et al. showed an increased frequency of EBV-specific CD69^+^ CD4^+^ T cells producing IFN-*γ* in SLE patients compared with controls. However, the frequency of EBV-specific CD69^+^ CD8^+^ T cells producing IFN-*γ* tended to be lower in SLE patients [34]. These differences might be due to the use of different stimulation periods (6 hours vs 24 hours in our study) or/and the specific EBV antigens used.

Similarly, Draborg et al. showed a significantly reduced number of activated T cells and IFN-*γ* production upon stimulation with EBNA 1 or EBV early antigen diffuse (EBV-EA/D) in SLE patients, suggesting the decreased control of EBV infection in these subjects [25]. Interestingly, even if the difference was not statistically significant, we observed a decreased T cell response in patients with detectable EBV DNA compared to those patients with undetectable EBV DNA, probably related to the reduced control of infection. A crucial role is exerted by immunosuppressive therapy. Indeed, among patients with detectable EBV DNAemia, significantly lower specific T cell activity was detected in the mhp-IS subjects in comparison with lp-IS.

A few studies [34–39] have investigated the role of HCMV infection in SLE pathogenesis, but its correlation with SLE has not been established [40]. According to some reports, the prevalence of HCMV infections in healthy subjects and SLE patients was similar [41, 42]. However, other studies have demonstrated that the prevalence of HCMV infection between the two groups is significantly different [35–37].

Larsen et al. did not find immune alterations in HCMV-specific T cell responses in SLE patients compared with controls [39]. Another study demonstrated that the frequency of HCMV-specific CD69^+^ CD4^+^ T cells producing IFN-*γ* and TNF-*α* was similar in SLE patients and healthy controls, while the frequency of HCMV-specific CD69^+^ CD8^+^ T cells producing IFN-*γ* and TNF-*α* was lower in SLE patients, although the difference was not statistically significant [34]. On the contrary, our results suggest a reduced HCMV-specific T cell response, in terms of CD4^+^ and CD8^+^ T cell IFN-*γ*-producing cells.

Analyzing HCMV-specific T cell response in SLE patients according to immunosuppression level, we do not have the evidence of the difference between lp-IS SLE patients and healthy HCMV-seropositive subjects, while mhp-IS patients showed a markedly reduced HCMV-specific T cell response if compared to lp-IS patients as well as to healthy HCMV-seropositive subjects. This could support the role of immunosuppressive treatment in the control of HCMV reactivation.

Interestingly, in our study, a significantly lower T cell response to the specific PHA antigen in SLE patients compared with healthy subjects was demonstrated, while other authors have not shown any differences [25, 38], suggesting that different antigens modify the type and the magnitude of the immune response. Patients with HCMV DNAemia have decreased control of infection, as suggested by the lower HCMV-specific T cell response in these patients, which is mainly related to immunosuppressive therapy.

In contrast with other studies [25, 39], we showed reduced EBV-specific CD4^+^ and CD8^+^ T cell responses in SLE patients treated with medium-high immunosuppression. Similarly, HCMV-specific CD4^+^ and CD8^+^ T cells had reduced IFN-*γ* production in medium-high immunosuppressed SLE patients. On the contrary, there was no difference in terms of PHA-specific T cell response between low and medium-high immunosuppressed SLE patients.

The role of immunosuppressive therapy appears to be crucial mainly to the response to EBV but also to HCMV-specific T cell response. Furthermore, a generally deficient immune response in SLE patients with respect to healthy controls was observed, supporting the hypothesis that SLE disease exerts a general immunosuppressive action regardless of the therapy. To corroborate this hypothesis, virus-specific T cell responses need to be analyzed in a larger number of patients, in order to stratify patients according to immunosuppressive status and iatrogenic risk factors.

## Figures and Tables

**Table 1:** Characteristics of study population.

	SLE patients (70)	Healthy subjects (50)	*P* value^∗^
Gender (female/male)	64/6	35/15	0.0032
Age (years, median (IQR))	46.5 (38.0-57.8)	44 (34.8-50.0)	0.1174
Serology			
EBV-seropositive	68	46	
EBV-seronegative	2	4	0.2331
HCMV-seropositive	56	35	
HCMV-seronegative	14	15	0.2795
DNAemia			
EBV DNA-positive	30	4	<0.0001
HCMV DNA-positive	3	0	0.2821
Viral load			
EBV DNA (copies/ml, median (IQR))	233 (100-656.3)	125 (62.5-225)	0.1895
HCMV DNA (copies/ml, median (IQR))	200 (100-1000)	Undetectable	
Immunosuppression			
lp-IS	25		
mhp-IS	45		
SLEDAI score			
<4	45		
≥4	25		

Demographic characteristics of SLE population and healthy controls are summarized in the table. (^∗^) *P* values were calculated using Fisher's exact test.

## Data Availability

The data used to support the findings of this study are available from the corresponding author upon request.
